# Normalized data may have limitations in determining the reliability of MVC measurements

**DOI:** 10.1038/s41598-025-20014-9

**Published:** 2025-09-25

**Authors:** Christoph Anders, Christin Alex, Max Herzberg, Lena Mader

**Affiliations:** Division of Motor Research, Pathophysiology and Biomechanics; Experimental Trauma Surgery, Department for Hand, Reconstructive, and Trauma Surgery, Jena University Hospital, Friedrich-Schiller-University Jena, Jena, Germany

**Keywords:** Maximum voluntary contraction, Trunk extension, Trunk flexion, Data normalization, Outcomes research, Laboratory techniques and procedures

## Abstract

Measurement data serve as an objective basis for scientific findings. Therefore, their reliability in repeated measurements is a crucial prerequisite. The repeatability of measurements is quantified using various reliability parameters. Often, data are subjected to normalization procedures to reduce inter-individual variability or to improve interpretability. In our study, we aimed to investigate the extent to which the application of normalization has impact on the determined reliability parameters. This was examined using the example of maximum force values for trunk extension and flexion. For this purpose, 85 healthy individuals (42 women) were subjected to maximum isometric force tests of the trunk muscles at two-weeks intervals. The calculated reliability and agreement parameters included the intraclass correlation coefficient (ICC), the standard error of measurement (SEm), the standard error of the mean (SEM), and the coefficient of variation of the method error (CVME). The ICC values consistently indicated good reliability for the original data (Extension: 0.889, Flexion: 0.882). Within the subpopulations of women and men, lower but still good ICC levels were observed (Women Extension: 0.671, Flexion: 0.826; Men Extension: 0.819, Flexion: 0.680). For the anthropometrically normalized force values, lower ICC levels were found for the entire group (Extension: 0.803, Flexion: 0.768) although the deviations in the subpopulations were different (Women Extension: 0.747, Flexion: 0.805; Men Extension: 0.852, Flexion: 0.642). Thus, normalization of measured values leads to varying deviations of calculated ICC levels compared to the original data, which is why the use of standardized data is not recommended for reliability calculations.

**Supplementary Information:**

The online version contains supplementary material available at 10.1038/s41598-025-20014-9.

## Introduction

In order to generate or expand knowledge, findings must be collected. Depending on the field of research, this is achieved with different instruments or via corresponding parameters. In psychological research, for example and therefore incomplete, subjective information on emotional state^1^, stress^2^, state of mind^3^ or quality of life^4^ are the main target variables. In life sciences, from biology to physiology, medicine, biomedicine, nutritional sciences and veterinary medicine (research disciplines are not listed completely), measurements of the corresponding so-called objective parameters form the basis of all research. These are then crucial for the identification of physiological parameters, which are used diagnostically, particularly with regard to their deviation from normal ranges. Furthermore, corresponding parameters are recorded and assessed over time, for example over periods of life or in the short term as part of therapeutic interventions^5^.

However, any diagnostic interpretation of data is tied to compliance with the basic quality criteria of measurements: Validity, reliability, and objectivity^6^. Related to this are the sensitivity and specificity of the measurement method^6^, which should ensure that changes are reliably recorded (sensitivity) and can be assigned to the cause as clearly as possible (specificity).

This rather general scientific-theoretical introduction has a direct impact on the possibility of interpreting the obtained data. Particularly in the case of physiological parameters, their suitability for diagnostic statements is determined, among other things, by whether these data are stable over uninfluenced periods of time or can be measured with appropriate repeatability. Any interpretation of changes depends on this. In the context of human studies, however, various external influences must be taken into account^7^. This is all the more true for studies that i) are subject to motivational influences^8^ and ii) show large inter-individual variability^9^. Force values determined during maximum voluntary contraction (MVC) can serve as an example^9,10^. These MVC values are used to diagnose individuals with respect to their physical performance, but also to monitor the success of training programs^11^. In sports science, MVC tests are still the gold standard for performance testing^12,13^. Corresponding data are collected and used for training management. However, whether such values are transferable to naive individuals is still the subject of scientific research and debate. In addition to motivational influences, the very large inter-individual variability of the data also plays an important role in their interpretation^14^. Ultimately, data that vary largely between individuals are difficult to interpret. This is where standardization procedures come into play—for the aforementioned maximum force, for example, to anthropometric parameters such as body weight^15,16^. This results in a significant advantage for the evaluation, as anthropometric standardization makes force data individually assessable on the one hand, and on the other hand, standardization should always lead to a reduction in inter-individual variability.

Although numerous studies have investigated MVC, its clinical relevance is limited^17–20^. In order to move MVC measurements beyond their traditional role in assessing training efficiency, we have chosen to focus on maximal force of the trunk muscles. The importance of adequate muscular force for maintaining spinal integrity is well established^21–24^. While the back muscles are naturally central to diagnostic evaluations in this context, the abdominal muscles also play a significant role. Specifically, the abdominal muscles biomechanically contribute to spinal stability by generating adequate intra-abdominal pressure^25^. This has been associated with back pain in cases of dysfunction^26,27^.

Consequently, functional testing of the abdominal muscles is an essential component of therapy monitoring within targeted training programs^28,29^. The role of either psychological^30–32^ and neuromuscular coordination factors^33,34^ in the development of acute and chronic back pain are not to be discounted; however, this study focuses specifically on performance-related aspects of trunk musculature.

To our knowledge, although maximum force performance is frequently examined, established normative values remain scarce^35^. This is particularly relevant to the functional interpretation of the considerable interindividual differences observed in pure force capacity. As previously mentioned, standardization appears to be a reasonable approach to enhance the individual assessment and interpretation of collected data.

In general and for practical purposes, the question therefore arises as to which values should be used for reliability measures. As explained, the usefulness of standardization procedures is certainly undisputed, as this means that interesting and practically relevant additional parameters, such as the determination of the physiological force reserve at maximum force, can be used more reasonable for diagnostic purposes than data without an anthropometric context.

Therefore, we investigated maximal force testing of the trunk musculature in a practically relevant measurement scenario, conducting repeated assessments in individuals who had no prior experience with such tests. The key target parameter was to directly compare reliability and agreement parameters between the non- normalized and anthropometrically normalized MVC data, as this has not been published yet. Secondary target parameter was the determination of the respective value ranges to ensure that the obtained data corresponds with data found in the literature.

## Methods

### Population

Participants were recruited through regional press announcements and electronic media. For this study, a total of 85 healthy individuals (42 women) aged 24 to 52 years were examined. The anthropometric data of the population can be found in Table 1. The data presented here are part of a larger study, partial results of which have already been published^36^.

**Table 1:** Anthropometric characteristics of the study cohort (at first measurement time point ).

	Age [years]	Height [cm]	Weight [kg]	BMI [kg/m^2^]
Female (n = 42)				
Mean (SD)	35.0 (8.4)	167.7 (7.1)**	65.6 (9.4)**	23.3 (2.9)*
Male (n = 43)				
Mean (SD)	34.9 (7.9)	179.3 (7.2)**	79.4 (10.4)**	24.7 (2.6)*

SD: standard deviation; BMI: body mass index; statistical differences female vs. male: * p < 0.05; ** p < 0.001.

Exclusion criteria for the study included age < 20 or > 55 years (to avoid influences of ongoing adolescence and also to avoid involution effects), active sports participation > 3 h per week (questioned during recruitment and again immediately prior to investigation), current pain in the spine or trunk region, and a history of spinal surgery. Additionally, all participants underwent a brief clinical-orthopedic screening examination (conducted by C.An.). Written informed consent for voluntary participation was obtained from all participants. The study was reviewed and approved by the Ethics Committee of Friedrich Schiller University Jena (2021–2373-BO, 2021-2373_1-BO). Thus, the study complies with ethical standards for research involving human participants and adheres to the current version of the Declaration of Helsinki.

### Investigation/data

#### Device

The subjects were positioned in a computerized trunk muscle testing and training device (CTT Centaur, BfmC Leipzig, Germany). Reliability of the data, detected with the device has been proven^37^. In the device, they stood in upright body position, secured from the hips downward while the upper body remained movable. A bar equipped with force sensors in the x- and y-directions was positioned over the participants’ shoulders, which captured the force data during the MVC tests (see below). All tests were performed in this device. The device can be tilted from 0° to 90°, while any rotational angle between − 180° and + 180° can be set. As the device tilts the subject completely, regardless of its position in space, the subject always remained in upright body position.

#### Determination of upper body weight

**Fig. 1:**
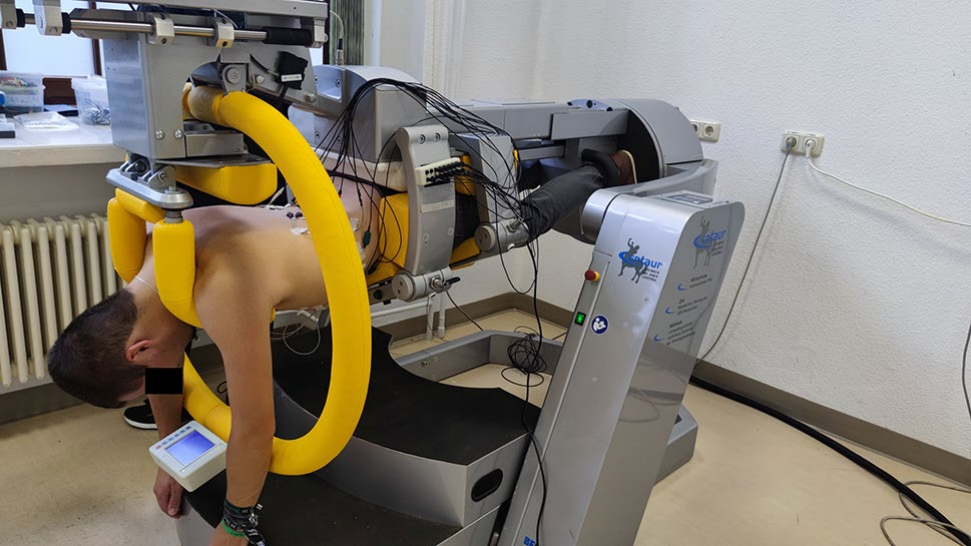
Subject positioned in the device while laying relaxed in 90° forward tilted position for determination of the upper body weight.

At the beginning of the examination, the upper body weight (UBW) was determined by tilting the device 90° forward (horizontal position), allowing the participants to relax into the shoulder bar (Fig. 1). Visual and tactile assessments of the back muscles ensured full relaxation of the back muscles. If residual contractions were detected, the subjects received appropriate feedback to help them relax completely. The measurement was carried out three times, and the highest reliable value was recorded to later determine the ratio of UBW to the maximal force (anthropometric normalization of force data). Immediately thereafter, the participants performed a series of defined isometric submaximal extension and flexion tests in the device. These data were analyzed for further research questions and simultaneously served as a warm-up before the maximal force tests.

#### Maximum voluntary contraction test

The isometric maximal force tests were conducted three times in flexion and also three times in extension direction, with a measurement duration of five seconds per trial and a five seconds rest period between repetitions. Participants were instructed to reach their maximal force within one second and maintain it for approximately three seconds. The first attempt was performed as a familiarization trial with approximately 50% of the self-estimated maximal force^35^, while the subsequent two trials were executed with maximal effort. The best of these two trials was used for further analysis. During the maximal force trials, verbal encouragement was consistently provided^38^. Testing always began with extension, followed by flexion. During the tests, subjects kept their arms crossed in front of their chests.

Subsequently, the force data were extracted from the analysis software of the CTT Centaur and included in the analysis. The entire examination (T1) was repeated in an identical manner after 14 days (T2).

### Analysis parameters

For the reliability analysis, both the measured force values and force values normalized to the upper body weight were used. The following reliability and agreement parameters were applied: the Intraclass Correlation Coefficient (ICC), the Standard Error of Measurement (SEm), the Standard Error of the Mean (SEM) and the Coefficient of Variation of the Method Errors (CVME). They are briefly explained below based on^36^.

#### Intraclass Correlation Coefficient (ICC)

We used the ICC as ICC (2,1)^39^ ( alternatively ICC (A,1)^40^) for comparing data from two measurement occasions. The settings for the calculation in SPSS (IBM, Chicago, USA) were as follows: single rater, absolute agreement, two-way mixed effects^40,41^. The ICC value can range from 0 to 1, with values close to 1 indicating excellent reliability and values below 0.5 indicating poor reliability^42^.

#### Standard error of measurement (SEm)

It represents the standard deviation (SD) of the mean of an infinitely repeated measurement (“true value”). However, this is practically unfeasible. Therefore, each individual result is considered the best estimate of the true value, with some sampling error. This sampling error can be assumed to be normally distributed with the corresponding SD. For two measurement occasions (m = 2), the SD of all observation points (m*n with n = 85 in the actual case) is multiplied by the square root of 1—ICC^43,44^. Since the SEm takes the ICC into account for its calculation, it depends directly on the level of the ICC and becomes smaller as the ICC increases. No normative values are available for the SEm.

#### Standard error of the mean (SEM)

The SEM provides information about the variance in repeated measurements. These means themselves have a mean, for which an SD can be calculated. This SD of such a distribution of means is called the SEM and indicates the distribution of random fluctuations in the estimation of the mean of several samples. Its boundaries correspond to the well-known limits of the SD, i.e., 68% within ± 1SEM and 95% within ± 2SEM. This allows for the definition of the uncertainty range of a measurement. Values outside this range, with the corresponding probability, are unlikely to be random fluctuations. The SEM is calculated as the quotient of the SD of the difference between the two measurement occasions and √n^45^ (n = 85) and is therefore a parameter of agreement.

#### Coefficient of variation of the method errors (CVME)

The CVME is the method error (ME) normalized to the mean of the two measurements, which is conceptually similar to the SEM but uses √2 in the denominator instead of √n for calculation. Its interpretation differs from that of the SEM and primarily indicates the systematic error of the measurement methodology. The values are presented as percentages due to the normalization, which facilitates their interpretation^44,46^. A review about reliability of ultrasound based determination of quadriceps femoris muscle thickness revealed a relative error of 6.5%^47^. Given that MVC measurements are subject to both external and particularly internal influences, we anticipated that the CVME would be approximately twice as high, around 13%. Values above 15% would therefore be considered as unacceptable, i.e. of low agreement.

##### Minimal detectable difference

We also calculated the minimum detectable difference (MDD) to identify the threshold of clinically relevant changes in the parameter expression^5,48,49^. This value is provided primarily for the sake of completeness, as it is mainly used in the clinical context to detect if actual changes in parameters over the course of a disease are to be considered relevant. It is calculated as the product of the SEm*z-score*√2, and is therefore directly dependent on the level of the ICC^49^.

### Statistics

First, a test for normality was conducted (Shapiro–Wilk). Since normal distribution was confirmed, the values from both days were compared using the t-test for dependent samples. A similar comparison, but for independent samples was made between the sexes. The respective effect sizes for dependent samples were calculated for all tests, i.e. dividing the averaged difference by its standard deviation^50^.

## Results

**Fig. 2:**
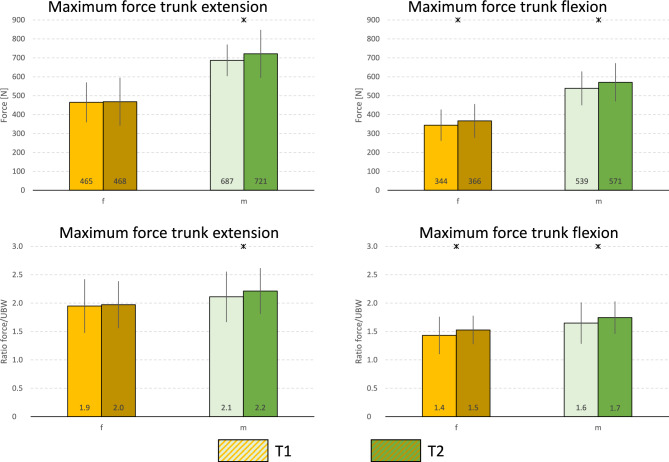
MVC force levels for trunk extension and flexion. Upper panel: originally measured force values Lower panel: anthropometrically normalized force values. Data are displayed as mean values ± SD. Asterisks indicate statistically significant differences between measurement times (p < 0.05).

For both sexes, statistically higher mean values were achieved at T2 except for the maximal extension in the female subgroup (Fig. 2). The respective effect sizes can be found in Table 2.

**Table 2:** Effect sizes for MVC T1 vs. T2 comparisons.

	Extension abs. force	Extension normalized	Flexion Abs. force	Flexion normalized
ALL SUBJECTS	0.263	0.225	0.451	0.463
WOMEN	0.058	0.061	0.487	0.468
MEN	0.492	0.503	0.442	0.454

The measured force values differed highly significantly between sexes, regardless of the time point. When analyzing the normalized data significance levels and also effect sizes decreased (Table 3).

**Table 3:** Between sex differences of MVC data.

	Extension abs. force	Extension normalized	Flexion Abs. force	Flexion normalized
*p-values*				
T1	< 0.001	0.102	< 0.001	< 0.01
T2	< 0.001	< 0.05	< 0.001	< 0.01
*Effect sizes*				
T1	1.908	0.358	2.266	0.736
T2	2.353	0.567	2.138	0.668

Reliability statistics across the entire group showed ICC values > 0.767. When examining the ICC levels separately by sex, generally lower values were found, with values > 0.641 observed (Table 4).

**Table 4:** Averaged values, as well as the results of the reliability calculations across all participants and by sex.

		MV	SD	CV	ICC (ICC low/ICC up)	SEm	SEM	CVME	MDD
*All subjects*									
Extension	Absolute force	587 N	159 N	27%	0.889 (0.804/0.945)	54.2 N	8.12 N	9.0%	150 N
	Normalized values	2.06	0.42	20%	0.803 (0.657/0.898)	0.20	0.03	9.4%	0.54
Flexion	Absolute force	456 N	132 N	29%	0.882 (0.811/0.947)	46.6 N	6.59 N	9.4%	129 N
	Normalized values	1.59	0.31	20%	0.768 (0.641/0.892)	0.16	0.02	9.2%	0.44
*Women*									
Extension	Absolute force	467 N	86 N	19%	0.671 (0.436/0.815)	54.0 N	11.9 N	11.7%	150 N
	Normalized values	1.96	0.43	22%	0.747 (0.552/0.860)	0.23	0.05	11.8%	0.63
Flexion	Absolute force	355 N	83 N	23%	0.826 (0.729/0.922)	36.1 N	7.23 N	9.3%	100 N
	Normalized values	1.48	0.33	22%	0.805 (0.695/0.911)	0.15	0.03	9.6%	0.43
*Men*									
Extension	Absolute force	704 N	122 N	17%	0.819 (0.720/0.919)	54.0 N	10.7 N	7.0%	150 N
	Normalized values	2.16	0.40	18%	0.852 (0.771/0.935)	0.16	0.03	6.6%	0.44
Flexion	Absolute force	555 N	88 N	16%	0.680 (0.506/0.843)	54.4 N	11.0 N	9.2%	151 N
	Normalized values	1.70	0.25	14%	0.642 (0.455/0.822)	0.16	0.03	9.0%	0.45

MV: mean value; SD: standard deviation; CV: coefficient of variation; ICC: Intraclass Correlation Coefficient; ICC low /up: ICC lower and upper borders (95% confidence interval); SEm: Standard Error of Measurement; SEM: Standard Error of the Mean; CVME: Coefficient of Variation of the Method Errors; MDD: minimal detectable difference (synonym: smallest real difference (SRD)) ^49^.

## Discussion

In the present study, MVC force values of the trunk for extension and flexion were analyzed for their reliability in both women and men, following a two-week measurement repetition. Since such values are often characterized by large interindividual variability and therefore are difficult to interpret without anthropometric reference, they are normalized accordingly. This allows for the effective representation of key metrics, such as physiological reserve, and enables statements about individual performance capacity. It is essential, of course, to ensure the reliability of such normalized data as well.

### MVC data

As expected, women exhibited significantly lower MVC values compared to men (see Table 4). This can be plausibly explained by the markedly different muscle mass between sexes^51,52^. This difference pertains not only to overall muscle mass but also suggests sex-specific variations in muscle fiber architecture. Although the proportions of type I and type II fibers are similar between women and men^53^, notable differences exist in fiber cross-sectional areas. Specifically, men display significantly larger functional cross-sectional areas of their type II fibers, a characteristic not observed in women^54^.

However, when normalizing the force data to UBW, the pronounced differences in absolute force output were substantially reduced (see Table 3). This convergence in normalized force values can be attributed to the significantly higher upper body weight in men. As such, normalized force values allow for a more accurate interpretation than absolute values^55^. Particularly, the derived physiological force reserve serves as an important diagnostic parameter for assessing physical performance capacity. The present data are suitable for use as general normative values for the investigated population and the applied test conditions.

Despite the improved interpretability of normalized maximal force data, the analysis clearly demonstrates that such data are not suitable for reliability analyses. Among others this is due to the intentionally reduced interindividual variability resulting from normalization.

It is also noteworthy that the recorded maximal force values improved in extension for men and in flexion for both sexes. Specifically, men showed increases of 4.9% in extension and 5.9% in flexion, while women improved their flexion force by 6.4%. These results demonstrate a maximum force gain that can be interpreted as a learning effect, despite ICC levels > 0.64 within the subgroups^56^. Interestingly, this effect was more pronounced for flexion than for extension. This may be due to the relatively unfamiliar nature of flexion exercises, whereas extension loads are more likely to be familiar. It can therefore be concluded that unfamiliar tasks are accompanied by learning effects upon repeated execution, which in turn improve performance^57^. However, the duration of such learning effects cannot be determined from the present data, and further targeted studies would be necessary to address this question. On the other hand, the also calculated MDD levels argue against these changes to interpreted as relevant, as their differences are all well below the determined MDD values.

Regardless of the reported values and observed sex differences, previous studies have demonstrated high reliability for non-standardized measurements of maximal trunk muscle force^58^. Furthermore, the values obtained in the present study are in good agreement of previously published reference data^35,59^.

### General statements according the ICC

The calculation algorithm for the primary reliability parameter, the ICC, has specific considerations that have to be taken into account. First, the correct model for its calculation should be selected. In the present case (Eq. (1)), this would be the ICC(2,1)^39^ or ICC(A,1)^40^.

$$ICC\left(\text{2,1}\right)=\frac{BMS-EMS}{BMS+\left(k-1\right)EMS+k(JMS-EMS)/n}$$

Equation (1): calculation of the ICC(2,1) according to^39^, BMS: between targets mean square (variance between subjects), EMS: estimated means square (residual variance), JMS: between judges mean square (variance between observations), k: number of observations, n: number of subjects.

The components of the equation may lead to confusion rather than being helpful without the appropriate mathematical background, which is why the influence of the equation’s components on the ICC will briefly be explained here. The so-called "between-subject variability" (BMS), or the variability of values between individuals, plays a crucial role in determining the final ICC value. When this variability increases, while all other parts of the equation remain constant the ICC will also increase, as it located in the numerator of the equation. At the same time, the repeatability—essentially the target measure of such calculations (JMS)—affects the ICC value. As expected, high variability between measurements leads to a decrease in the ICC value.

Additionally, there is another influencing factor, the so-called residual variability (EMS), which results from the spread of values per individual and the differences between observation time points. Furthermore, sample size plays an important role as well— the more individuals considered, the higher the ICC values tend to be. Therefore, it is difficult to predict the impact that normalization of the force values will have on the ICC and, consequently, on the other reliability parameters.

### Effect of the normalization

The goals of any applied normalization procedure can be divided into i) reducing the interindividual variance of the values being assessed or ii) relating the values to individual characteristics, i.e. the applied anthropometric normalization for a more meaningful and therefore improved interpretation of data. As i) inevitably leads to a reduction of the ICC ii)'s consequences on ICC levels are not obvious. Therefore, the global means are presented in Table 3, and the dispersions per subgroup and measurement time point are shown in Fig. 2. However, it is specifically observed for the force data analyzed here that normalization to the upper body weight improves the interpretability of the maximal force values significantly but does not lead to a consistent reduction in the variation between individuals. This ca most clearly be seen in the variation coefficients, which are also displayed: here, the SD is expressed as a percentage relative to the mean value level. It can be observed that normalization resulted in an increase of the variability for extension in the female participants, but a decrease for flexion. In the male participants, the opposite occurred: normalization led to a reduction of the variability for flexion and an increase for extension. Seemingly paradoxically, these changes in interindividual variability of the values were accompanied by corresponding changes in ICC levels. Thus, the otherwise meaningful anthropometric normalization led to an ambiguous distortion of the ICC values, making them no longer unequivocally interpretable. In a transferable sense, these considerations also apply to the standard error of measurement (SEm), as the ICC is part of its calculation.

### Further influencing factors: value level and sample size

The mean MVC level clearly differed between women and men, with the sex differences in the normalized values decreasing significantly in magnitude and, consequently, in the clarity of these differences. This further highlights the relevance of anthropometric normalization, which demonstrates a strong convergence between the sexes in the force ratio between MVC and UBW. Through anthropometric normalization, the values of the physiological reserve approximate despite clear force differences, so that for extension, at least at T1, no sex differences were detectable (Table 3). In other words, this means that although there are large differences in absolute force levels between both sexes, these are much smaller in relation to the physiological force reserve and tend to be negligible. This effect becomes particularly evident when considering the respective effect sizes (see Table 3), which, despite statistical comparisons remained significant, decreased to moderate levels^60^. Another effect of MVC levels, independent of their influence on the ICC, was observed for the standard error of the mean (SEM), which, in turn, directly affected the coefficient of variation of the measurement error (CVME). At comparable repeatability, the values of the differences between the repeated measurements showed a direct correlation, initially leading to a corresponding change in SEM. However, this change was inversely correlated with sample size. Consequently, the effects of the individual calculation components on SEM are complex and, therefore, difficult to interpret.

In contrast, the influence of value level and sample size on CVME is less complex, as sample size does not affect its calculation. Thus, at comparable repeatability, no systematic changes in CVME are expected across different MVC levels. This was confirmed by the data of the present study: while CVME values showed only minor differences between the original force values and the normalized values, only the distinctly different reliability levels for flexion and extension in women and men were associated with corresponding changes in CVME values.

## Limitations

The investigation was conducted using a specific test and training device (CTT Centaur, BfmC Leipzig, Germany), which limits the mobility of the upper body in an upright position. Since the bar for force measurement was positioned cranially over the shoulders, it cannot be excluded that forces were also produced in the cranial direction due to possible contact during the maximum force measurement. Since investigators were aware of this potential error in tasks execution, they closely monitored the performance of the exercises. In case of implausibly low values, instructions were repeated, the exercise practiced with submaximal force, and finally the MVC test was repeated.

Another limiting factor arises from the studied population. Participants were individuals who where not engaged in intensive sports activities in their leisure time, and thus had little to no experience with maximum force exercises. The systematically higher maximum force values at time T2 suggest a habituation or learning effect^56^. Although this is a commonly observed effect in practice when investigating naïve persons, references to it in the literature are unfortunately sparse. We found only one specific study, but this relates to muscle activation, which was not reported here^61^.

To compensate for this, the so-called familiarization trial with submaximal force was always conducted beforehand^35^. Only the two remaining trials were included in the analysis.

Another influencing factor for maximum force measurements arises from motivation of the participants and the experimental conditions in terms of verbal encouragement. Since it is known that verbal encouragement positively affects performance^38,62^, all participants were encouraged accordingly to ensure this effect was consistent for everyone.

## Summary

Reliability analyses should always be conducted using non-normalized original values. Any normalization of measurement values, which may be meaningful for diagnostic interpretation or for assessing group variability, influences reliability metrics in a complex and thus difficult-to-trace manner. Consequently, normalized data are not suitable for deriving reliability indices. This was demonstrated in the present study using the example of MVC measurements of the trunk for flexion and extension.

## Conclusion

Maximal force measurements of trunk muscles show good intersession reliability. However, when analyzed by sex, they decrease to moderate levels for women in extension and for men in flexion. With respect to force levels men demonstrate approximately 1.5 times higher absolute force values than women, independent of force direction. This difference, however, reduces to approximately 1.1 when anthropometrically normalized values are used. In general, anthropometrically normalized force values enhance comparability of MVC data. However, the impact of normalization on reliability values is variable and can lead to both poorer and better reliability levels. These deviations are not consistent. Therefore, for reliability studies, original (non-normalized) values should always be used to avoid unsystematic distortions of reliability outcomes.

## Supplementary Information

Supplementary Information.

## Data Availability

All data generated or analyzed during this study are included in this published article and its supplementary information files.
